# Supplementary material for: Numerical investigation of module-level inhomogeneous ageing in lithium-ion batteries from temperature gradients and electrical connection topologies
Source: Commun Eng. 2024 Jun 25;3:89. doi: 10.1038/s44172-024-00222-3 (PMC11199702; doi:10.1038/s44172-024-00222-3)
Supplement: Supplementary file 2 — Supplementary Information [file 44172_2024_222_MOESM2_ESM.pdf]

Supplementary Information

Numerical investigation of module-level inhomogeneous ageing in lithium-ion batteries from temperature gradients and electrical connection topologies

Haosong He[1], Ashley Fly[2], Edward Barbour[1], Xiangjie Chen[1]*

1 Centre for Renewable Energy Systems Technology (CREST), Wolfson School, Loughborough University, Holywell Park, Loughborough, LE11 3GR, Leicestershire, UK.

2 Department of Aeronautical and Automotive Engineering, Loughborough University, Loughborough, LE11 3TU, Leicestershire, UK.

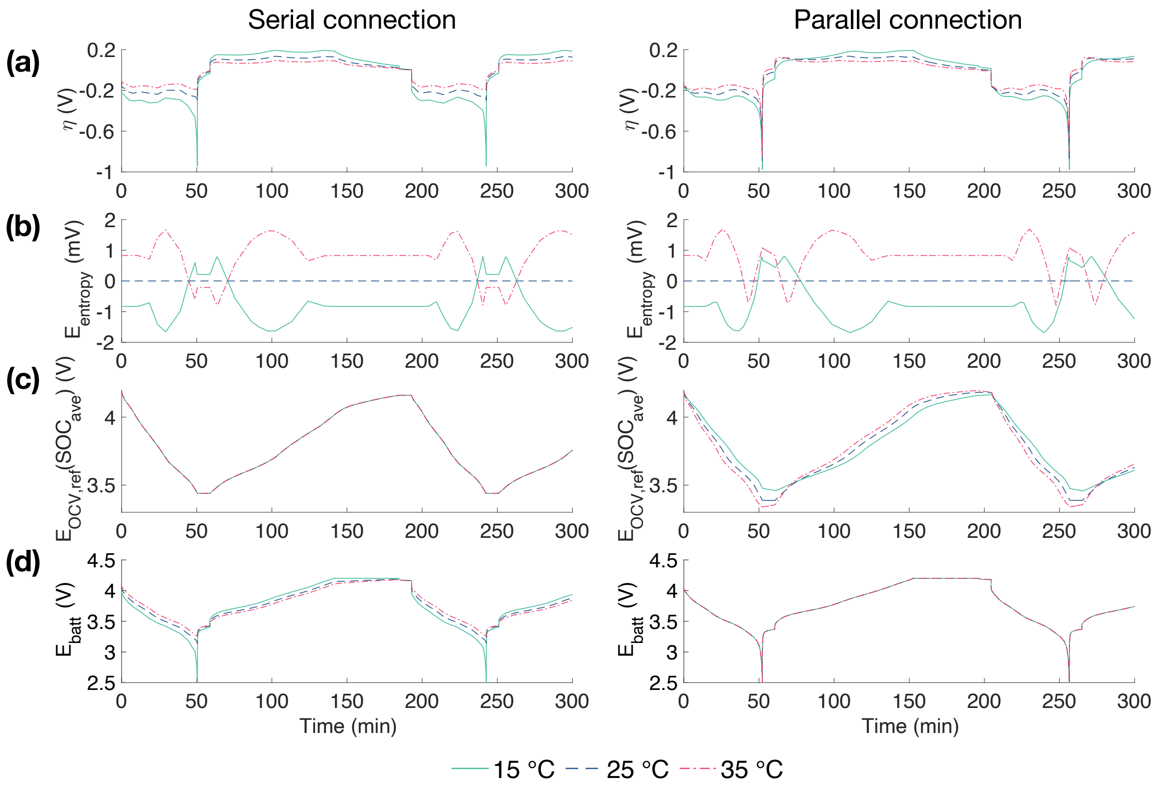

Figure S1: Comparison of the variance in electrochemical parameters due to temperature difference for serial and parallel connections. (a) Total overpotential. (b) Entropy voltage. (c) Open circuit voltage at the reference temperature. (d) Terminal voltage.

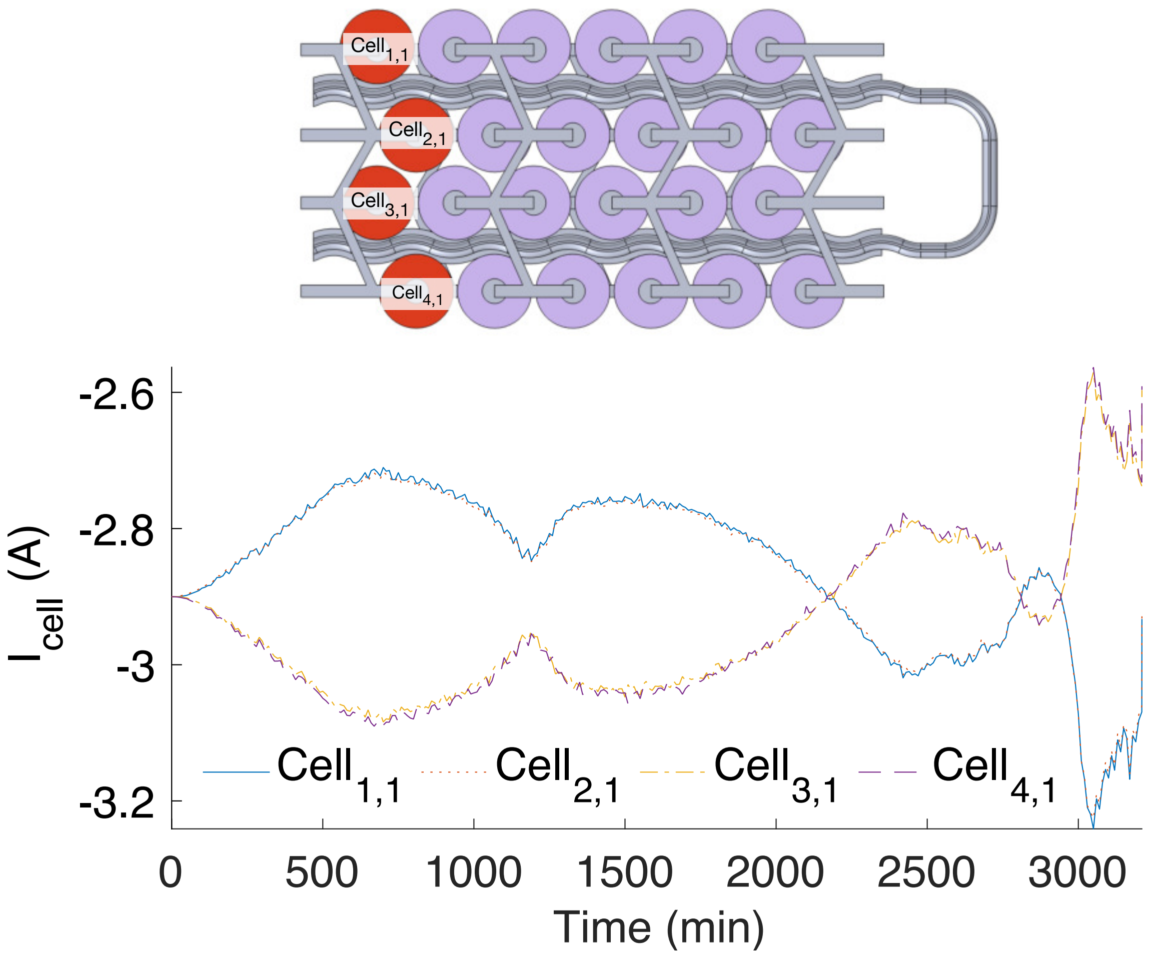

Figure S2: Current distribution of parallel-connected sub-module 1 (P1) for the straight design.

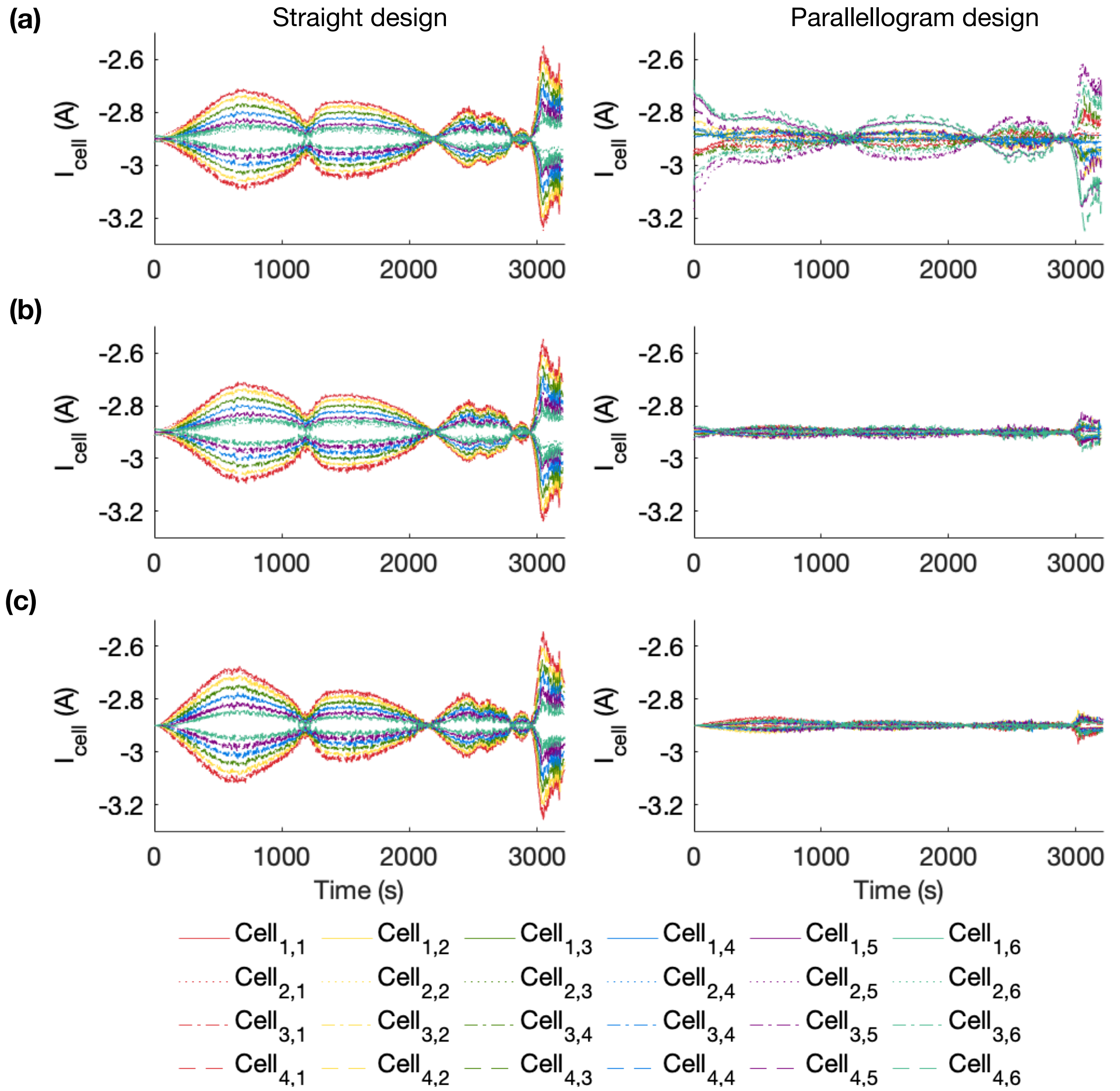

Figure S3: Comparison of the variance in current distribution for different interconnection materials. (a) Steel, $\sigma = 4.032 \times 10^6$ S m^{-1}. (b) Aluminium, $\sigma = 3.774 \times 10^7$ S m^{-1}. (c) Hypothetical super-low resistance, $\sigma = 1 \times 10^{12}$ S m^{-1}.

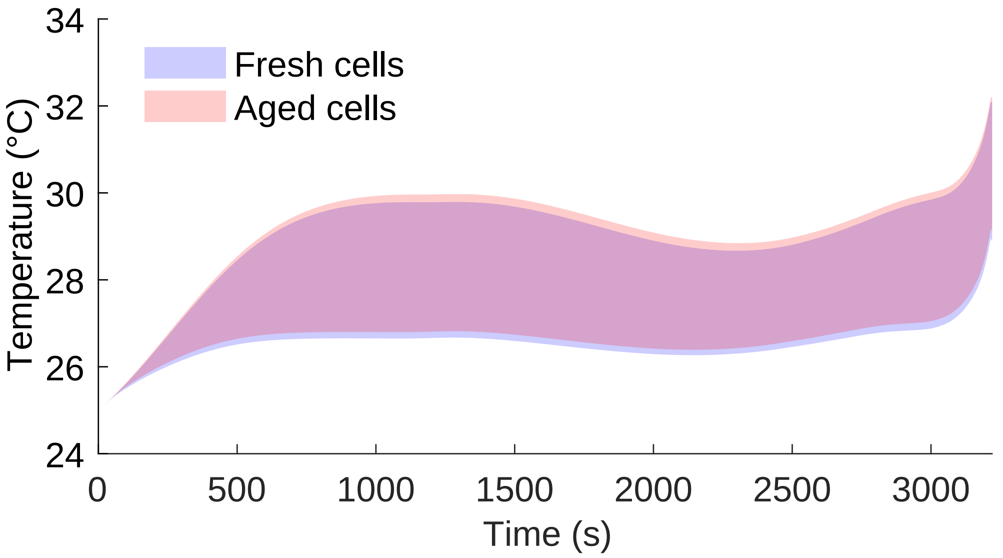

Figure S4: Comparison of temperature distribution for the fresh and aged cells for the straight connection topology.

Table S1: NCR18650PF cell specifications [1].

Material	$Li(NiCoAl)O_2$/Carbon
Capacity	2.9 Ah
Min/Max voltage	2.5 V/ 4.2 V
Charge temperature	0 - 45 °C
Discharge temperature	-20 - 60 °C
Storage temperature	-20 - 50 °C

Table S2: Variation of the entropy coefficient with SOC [2].

SOC_{ave}	$\partial E_{OCV}(SOC_{ave})/\partial T$ (mV K^{-1})
0.9	0.083
0.8	0.083
0.7	0.065
0.6	0.143
0.5	0.169
0.4	0.141
0.3	0.058
0.2	-0.082
0.1	0.069

Table S3: Symbols used in LSPM and ageing model.

Symbol	Property	Unit	Value
E_{act}	Activation energy for f_T	kJ mol^{-1}	Fitted
$E_{act}^{\eta_{ohm,1C}}$	Activation energy for $\eta_{ohm,1C}$	kJ mol^{-1}	Fitted
$E_{act}^{J_0}$ (kJ mol^{-1})	Activation energy for J_0	kJ mol^{-1}	Fitted
E_{act}^{τ}	Activation energy for τ	kJ mol^{-1}	Fitted
E_{batt}	Terminal voltage	V	Calculated
$E_{entropy}$	Entropy voltage	V	Calculated
$E_{OCV,ref}(SOC_{ave})$	Open circuit voltage at reference temperature	V	Calculated
$E_{OCV}(SOC_{ave}, T)$	Open circuit voltage	V	Calculated
$\overline{E_{OCV}}(T)$	Average open circuit voltage	V	Calculated
E_{offset}	Offset potential	V	Fitted
F	Faraday's constant	C mol^{-1}	96485
f	Total ageing factor	C mol^{-1}	Calculated
f_{aged}	Ageing history factor	-	Calculated
f_E	Voltage ageing factor	-	Calculated
f_I	Current ageing factor	-	Calculated
f_T	Temperature ageing factor	-	Calculated
G	Decelerating ageing factor	-	Fitted
H	Cycling capacity loss factor	-	Fitted
I_{1C}	1C current	A	11.6
I_{batt}	Applied current	A	-
I_{loss}	Parasitic SEI current	A	Calculated
J_0	Dimensionless exchange current	-	Fitted
N_{shape}	Particle dimension number	-	3
Q_{batt}	Rated capacity	Ah	2.9
$Q_{batt,0}$	Initial capacity	Ah	-
Q_{loss}	Capacity loss	Ah	Calculated
R	Molar gas constant	J $mol^{-1}K^{-1}$	8.1345
R_{ohm}	Ohmic resistance	Ω	Calculated
S	Local SOC in particle	-	Calculated
SOC_{ave}	Average SOC	-	Calculated
t	Time	s	-
T	Temperature	℃	-
T_{ref}	Reference temperature	℃	25
X	Dimensionless spatial variable	-	0-1
α	Transfer coefficient	-	Fitted
η	Total overpotential	V	Calculated
η_{act}	Activation overpotential	V	Calculated
η_{conc}	Concentration overpotential	V	Calculated
η_{ohm}	Ohmic overpotential	V	Calculated
$\eta_{ohm,1C}$	Ohmic overpotential at 1C current	V	Fitted
τ	Time constant	s	Fitted
τ_{loss}	Calendar ageing time constant	s	Fitted

Fitted: The fitted values are derived in Section 'Parameterisation'.

Calculated: The calculated values are derived based on the fitted values.

Table S4: Values of properties, specifications parameters of the battery module used in heat transfer model.

Property	Symbol	Unit	Value	Source
Cell				
Density	ρ_{batt}	kg m^{-3}	2734	[3]
Heat capacity	$C_{p,batt}$	J kg^{-1}K^{-1}	830	[3]
Axial thermal conductivity	$k_{batt,a}$	W m^{-1}K^{-1}	13.35	[4]
Radial thermal conductivity	$k_{batt,r}$	W m^{-1}K^{-1}	0.78	[3]
Initial temperature	T_0	°C	25	-
Coolant: deionised water				
Density	ρ_{cool}	kg m^{-3}	997	-
Heat capacity	$C_{p,cool}$	J kg^{-1}K^{-1}	4186	-
Thermal conductivity	k_{cool}	W m^{-1}K^{-1}	0.624	-
Dynamic viscosity	μ_{cool}	J kg^{-1}K^{-1}	1.002	-
Inlet velocity	$\vec{v}_i$	m s^{-1}	0.006	-
Cooling pipe: aluminium				
Density	ρ_{pipe}	kg m^{-3}	2700	-
Heat capacity	$C_{p,pipe}$	J kg^{-1}K^{-1}	900	-
Thermal conductivity	k_{pipe}	W m^{-1}K^{-1}	205	-
Busbar: steel AISI 4340				
Density	ρ_{bus}	kg m^{-3}	7850	-
Heat capacity	$C_{p,bus}$	J kg^{-1}K^{-1}	475	-
Thermal conductivity	k_{bus}	W m^{-1}K^{-1}	44.5	-
Electrical conductivity	σ_{bus}	S m^{-1}	1×10^{12}	Modified

Supplementary References

[1] Kollmeyer, P. Panasonic 18650pf li-ion battery data. *Mendeley Data* **1** (2018).

[2] Lu, Z. *et al.* A comprehensive experimental study on temperature-dependent performance of lithium-ion battery. *Applied Thermal Engineering* **158**, 113800 (2019).

[3] Murashko, K. A., Pyrhönen, J. & Jokiniemi, J. Determination of the through-plane thermal conductivity and specific heat capacity of a li-ion cylindrical cell. *International Journal of Heat and Mass Transfer* **162**, 120330 (2020).

[4] Jiang, Y., Huang, J., Xu, P. & Wang, P. Axial and radial thermal conductivity measurement of 18,650 lithium-ion battery. *Journal of Energy Storage* **72**, 108516 (2023).
